# Modeling the relationship between estimated fungicide use and disease-associated yield losses of soybean in the United States II: Seed-applied fungicides vs seedling diseases

**DOI:** 10.1371/journal.pone.0244424

**Published:** 2020-12-28

**Authors:** Ananda Y. Bandara, Dilooshi K. Weerasooriya, Shawn P. Conley, Tom W. Allen, Paul D. Esker

**Affiliations:** 1 Department of Plant Pathology and Environmental Microbiology, Pennsylvania State University, University Park, Pennsylvania, United States of America; 2 Department of Agronomy, University of Wisconsin-Madison, Madison, Wisconsin, United States of America; 3 Delta Research and Extension Center, Mississippi State University, Stoneville, Mississippi, United States of America; Julius Kuhn-Institut, GERMANY

## Abstract

Use of seed-applied fungicides has become commonplace in the United States soybean production systems. Although fungicides have the potential to protect seed/seedlings from critical early stage diseases such as damping-off and root/stem rots, results from previous studies are not consistent in terms of seed-applied fungicide’s ability to mitigate yield losses. In the current study, the relationship between estimated soybean production losses due to seedling diseases and estimated seed-applied fungicide use was investigated using annual data from 28 soybean growing states in the U.S. over the period of 2006 to 2014. National, regional (northern and southern U.S.), state, and temporal scale trends were explored using mixed effects version of the regression analysis. Mixed modeling allowed computing generalized R^2^ values for conditional (*R*^2^_GLMM(*c*)_; contains fixed and random effects) and marginal (*R*^2^_GLMM(*m*)_; contains only fixed effects) models. Similar analyses were conducted to investigate how soybean production was related to fungicide use. National and regional scale modeling revealed that *R*^2^_GLMM(*c*)_ values were significantly larger compared to *R*^2^_GLMM(*m*)_ values, meaning fungicide use had limited utility in explaining the national/regional scale variation of yield loss and production. The state scale analysis revealed the usefulness of seed-applied fungicides to mitigate seedling diseases-associated soybean yield losses in Illinois, Indiana, North Carolina, and Ohio. Further, fungicide use positively influenced the soybean production and yield in Illinois and South Dakota. Taken together, use of seed-applied fungicide did not appear to be beneficial to many of the states. Our findings corroborate the observations made by a number of scientists through field scale seed-applied fungicide trials across the U.S and reiterate the importance of need base-use of seed-applied fungicides rather than being a routine practice in soybean production systems.

## Introduction

Soybean [*Glycine max* (L.) Merr.] is among the most economically important crops in the United States, presently the world’s largest soybean producer (United States Department of Agriculture, World Agricultural Production Report 2019). In 2019, more than 30 million hectares in the U.S. were under soybean cultivation with a total production of approximately 97 million metric tons (United State Department of Agriculture—National Agricultural Statistics Service: USDA-NASS). Soybean production is often challenged by multiple factors such as inclement weather, nutrient-poor soil, and yield-limiting weeds, insects, and diseases [1, 2]. Various diseases can have deleterious effects on soybean yield, affecting both grain quantity and quality. In the U.S., approximately 11% of soybean yield is estimated to be lost annually due to various diseases [3]. Between 1996 and 2016, the estimated total economic losses due to soybean diseases in the U.S. was $95 billion [4]. Among various yield-limiting disease categories, seedling diseases (including root diseases) are of great importance. Even though an array of soilborne pathogens can cause soybean seedling and root diseases, the major ones include species of *Fusarium* and *Rhizoctonia solani* from the kingdom Fungi and the oomycetes *Pythium* and *Phytophthora* from the kingdom Stramenopila [5]. In addition, seed-associated organisms that generally result from the environment encountered during the prior season, but more broadly considered to be the result of multiple species of *Phomopsis* can also cause seedling disease-associated issues by reducing germination and producing poor stands. Seedling diseases are typically characterized by lesions on roots and pre- and post-emergence damping-off [5–7]. Decreased seedling vigor can occur when the infections are not severe yet result in damaged root systems ultimately leading to reduced yield [8]. In general, poor plant and poor stand establishment are the major causes behind yield losses due to seedling diseases in soybean [6]. Taken together, the organisms that cause seedling diseases are considered a complex as it is oftentimes difficult to determine which organism(s) are involved.

Seedling diseases can cause substantial soybean yield/economic losses. For example, the seedling diseases as a group were estimated to cause losses of approximately 1.6 million metric tons in 2014, which was second only to losses associated with the soybean cyst nematode [9]. Pathogenic organisms within the group broadly classified as the Oomycetes (*Pythium* and *Phytophthora*) have previously been estimated to reduce soybean yield in the U.S. by 0.68 million metric tons annually [10]. In the northern U.S., *Phytophthora sojae* has been estimated to cause $200 million in annual losses to soybean production, and worldwide causes approximately $1–2 billion in losses [11]. As per Bandara et al. [4], the total economic loss due to soybean seedling diseases in the U.S. between 1996 and 2016 was estimated at $17.6 billion which accounted for approximately 18.8% of the total economic losses due to all reported diseases.

In the U.S., there has been a general increasing trend in soybean yield losses as a result of seedling diseases [10, 12, 13]. The increased incidence of oomycete-related diseases could be attributed to lack of commercial soybean cultivars resistant to *Pythium* spp., ability of *Phytophthora sojae* pathotypes to overcome existing Rps resistance genes, changes in cultural practices used by growers such as earlier planting dates and reduced or minimum tillage, precision planting practices that tend to place seed in the same spot within the furrow between seasons, and changes in precipitation patterns such as greater rainfall in the spring and early summer [14, 15]. Moreover, resistant cultivars are not available against the specific organisms that cause seedling diseases broadly defined as damping-off [16]. Therefore, three methods have been historically used to manage these seedling diseases include crop rotation, tillage, and seed-applied fungicides. However, the wide host ranges of *Pythium* spp. as well as *R*. *solani* may limit the effectiveness of crop rotation [17, 18]. Moreover, with the changes in crop rotation practices, seedling diseases caused by the major soilborne pathogen groups are becoming more common [19]. As such, although crop rotation can be a useful strategy to control soil inhabiting plant parasitic nematodes and certain soilborne fungal pathogen species that belong to genera such as *Verticillium*, *Fusarium*, and *Alternaria*, it’s use can be limited in fields where *Phythium* spp. and *Rhizoctonia solani* are more problematic. Therefore, the use of seed-applied fungicides has become an important practice to manage seedling diseases in soybean. In fact, anecdotal evidence indicates that more than 50% of soybean seeds was treated with at least one fungicide in 2012 [20]. Farmers used to purchase and apply fungicides themselves or in close cooperation with applicators. However, purchase of fungicide-treated seeds have become the common practice nowadays.

Some of the more commonly used seed-applied fungicides include metalaxyl/mefenoxam (Phenyl Amides: negatively affect nucleic acids synthesis by acting as RNA polymerase I inhibitors) and fludioxonil (Phenyl Pyrroles: inhibits transport-associated phosphorylation of glucose, affect signal transduction). In general, these seed-applied fungicides reduce fungal/oomycete spore germination as well as mycelial growth [21]. In addition to the aforementioned fungicides, azoxystrobin, pyraclostrobin, and trifloxystrobin (quinone-outside inhibitors (QoI): disruption of electron transport chain, preventing ATP synthesis) are also used as seed-applied fungicides because they have broad spectrum activity against numerous seed and soilborne fungi. However, members of the QoI class of fungicides are most notably recognized as foliar fungicides. Seed-applied fungicides are generally applied in the form of multiple products (a.i.) since normally a single organism is not responsible for seedling diseases and soilborne organisms can work as a complex to reduce stand establishment.

The protective effect of seed-applied fungicides on seedling disease-mediated soybean stand and yield protection have previously been reported [19, 22–28]. The economic benefits of using seed-applied fungicides appear to be conditional upon factors such as commodity price, environment, quality of planting seed, as well as cultivar [19, 20, 29, 30]. However, the one economic benefit that cannot go unstated is the benefit that seed-applied fungicides provide by reducing the likelihood of a replant situation. Soybean farmers rely on seed-applied fungicide products to reduce stand losses that may occur as a result of catastrophic disease. Ideally, as a means of reducing the increased costs associated with herbicide trait technology fees and obtaining the best genetic offerings, soybean farmers attempt to offset those costs by reducing seeding rates. Therefore, seed-applied fungicides attempt to maximize the surviving stand with the first planting. However, many landscape level (location specific) studies have failed to demonstrate the ability of seed-applied fungicides to reduce the seedling disease-associated soybean yield losses [28, 31, 32].

Our companion paper [33] explored the relationship between estimated foliar fungicide use and soybean yield losses due to foliar diseases at national, regional, and state level using long term data. A joint analysis considering both foliar and seed-applied fungicides and associated yield loses was not performed due to a number of reasons. Although the agglomeration of both foliar and seed-applied fungicides under the central theme of “pesticides” seems technically sound, any conclusions derived from such an analysis are bound to be made about “pesticides” as a whole, on their ability to reduce yield losses due to foliar and soilborne fungal pathogens. Nonetheless, it should be noted that farmers make decisions on the use of foliar and seed-applied fungicide categories independently. As such, it is apparent that results generated through a joint analysis and inferences/conclusions drawn from such results are less meaningful from the actual production standpoint. Instead, partitioning of the pesticides into foliar and seed-applied categories is appropriate to ask more biologically relevant and topic specific research questions, recognizing that it is not possible to make any conclusions on the causation of such relationships since it is not possible to partition the effect of foliar and seed-applied fungicides on yield losses in the databases that we have available. Given such considerations, foliar and seed-applied fungicides were analyzed separately with appropriate statistical approaches (i.e. generalized R^2^, see methods section) to explore the relationship between estimated foliar/seed-applied fungicide use and soybean yield losses in our two companion papers.

Although numerous field experiments based on seed-applied treatments have been conducted and reported at a landscape level (i.e., location specific), a more comprehensive analysis with long term historical data to reveal the relationship between estimated seed-applied fungicide use and soybean yield losses as a result of seedling diseases is currently lacking. Therefore, our objectives for this study were to (i) investigate long-term seed-applied fungicide use patterns, (ii) investigate the relationship between seed-applied fungicide use and yield losses due to seedling diseases, and (iii) investigate the relationship between seed-applied fungicide use and soybean production/yield in the U.S. at national, regional, and state levels.

## Materials and methods

### Fungicide use, target diseases, and states considered

Annual state-level soybean seed-applied fungicide use estimates from 2006 to 2014 were acquired from the Pesticide National Synthesis Project webpage (https://water.usgs.gov/nawqa/pnsp/usage/maps/county-level/StateLevel/HighE stimate_AgPestUsebyCropGroup92to16.txt). The fungicide use data are presented in kg of each active ingredient (a.i.) in this source. Note that the database contains actual fungicide use estimates but not amounts that were sold. A much broader explanation of the associated seed-applied fungicide products contained within the report can be obtained by reading Thelin and Stone [34]. To compute fungicide use in units of grams per hectare, the amount provided in the database (in kg) was first converted to grams (g). For individual states, the soybean area harvested was retrieved from the USDA-NASS database for each given year (https://quickstats.nass.usda.gov). Fungicide use values (in g) were then divided by the respective harvested hectarage to determine the fungicide concentration in grams of fungicide per harvested hectare (here after mentioned as g/ha). The specific fungicide a.i.’s considered for this analysis included fludioxonil, metalaxyl, and mefenoxam. Due to difficulties in extracting the information as it relates to the use patterns of the QoI fungicides as seed treatments, we did not focus on the QoIs as seed treatments. Fusarium wilt (*Fusarium* spp.), Phytophthora root and stem rot (*Phytophthora sojae*), and seedling diseases caused by *Fusarium*, *Phomopsis* spp., *Pythium*, and *R*. *solani* were the target disease causing organisms for the seed-applied fungicide products considered.

The time period selected (from 2006 to 2014) was based upon the availability of fungicide use data, which spanned 28 soybean growing states (Alabama, Arkansas, Delaware, Florida, Georgia, Illinois, Indiana, Iowa, Kansas, Kentucky, Louisiana, Maryland, Michigan, Minnesota, Missouri, Mississippi, Nebraska, North Carolina, North Dakota, Ohio, Oklahoma, Pennsylvania, South Carolina, South Dakota, Tennessee, Texas, Virginia, and Wisconsin). We also classified the fungicide use data based on region where northern states considered for this study included Illinois, Indiana, Iowa, Kansas, Michigan, Minnesota, Nebraska, North Dakota, Ohio, Pennsylvania, South Dakota, and Wisconsin and southern states included Alabama, Arkansas, Delaware, Florida, Georgia, Kentucky, Louisiana, Maryland, Mississippi, Missouri, North Carolina, Oklahoma, South Carolina, Tennessee, Texas, and Virginia. The region (northern and southern U.S.) classification was based on two groups of soybean pathologists that have continued to compile disease loss estimate data on an annual basis as part of their Extension efforts and comprising the Southern Soybean Disease Workers and NCERA-137 (North Central Extension and Research Activity for Soybean Diseases).

### Estimation of soybean production/yield losses

The historical statewide annual soybean production loss (MT) data due to seedling diseases were acquired from soybean researchers and Extension specialists in each of the 28 soybean growing states mentioned above. See our companion paper [33] for details on the methodology adopted to compute soybean production loss data. We used the soybean production loss data for the same periods where seed-applied fungicide data were available (i.e. 2006–2014). To compute the per hectare yield loss due to a given disease within a given year, the total statewide annual soybean production loss (MT) due to that disease was divided by the respective statewide harvested hectarage (here after mentioned as Kg/ha).

### Computation of the relationship between yield losses and fungicide use

In order to model the relationship between yield losses and fungicide use at national (across 28 states and 9 years) and regional scales (Northern U.S. = across 12 states and 9 years, Southern U.S. = across 16 states and 9 years), we employed a linear mixed model version of the regression analysis [35]. Both null and full models were fitted with the assumed normal distribution for the response variable, yield loss. As yield losses and fungicide use data were classified by year and state, they were considered as random factors in null and full models while ‘fungicide use’ was specified as a fixed factor in the full linear model. Moreover, a quadratic model was also fitted by including the square term of fungicide use as a fixed factor. The models fitted were as follows.

Null model (contains only random factors):

$$L_{jk}=\beta_{0}+S_{j}+Y_{k}{+e}_{jk}$$

Full linear model (contains random and fixed factors):

$$L_{jk}=\beta_{0}+\beta_{1}F_{jk}+S_{j}+Y_{k}+e_{jk}$$

Full quadratic model (contains random and fixed factors):

$$L_{jk}=\beta_{0}+\beta_{1}F_{jk}+{\beta_{2}F_{jk}^{2}+S}_{j}+Y_{k}+e_{jk}$$

where, *L*_*jk*_ = soybean yield loss due to seedling diseases from j^th^ state in k^th^ year; *S*_*j*_ = random effect of the j^th^ state; *Y*_*k*_ = random effect of the k^th^ year; *F*_*jk*_ = seed-applied fungicide use from j^th^ state in k^th^ year; *F*^*2*^_*jk*_ = quadratic term for the seed-applied fungicide use from j^th^ state in k^th^ year; *β*_*0*_ = intercept; *β*_*1*_ = slope related to the seed-applied fungicide use from j^th^ state in k^th^ year; *β*_*2*_ = slope related to the quadratic term of the seed-applied fungicide use from j^th^ state in k^th^ year; e_ij_ = error.

According to the approach established by Nakagawa and Schielzeth [35], conditional R^2^ [*R*^*2*^_GLMM(*c*)_; fixed and random effects] and marginal R^2^ [*R*^*2*^_GLMM(*m*)_; fixed effects] versions of the full model were computed to assess the relative contribution of (fixed + random) and (fixed) factors to the observed yield loss variation. Akaike and Bayesian information criteria were computed with maximum likelihood (ML) specification while restricted maximum likelihood (REML) specification was used to estimate variance components. Models were fitted to examine the relationship between total soybean production loss (1,000 MT) and total seed-applied fungicide use (MT), as well as total yield loss per unit area (kg/ha) and total fungicide use per unit harvest area (g/ha). Similar analyses were conducted to investigate the relatedness of soybean production and yield with fungicide use. The packages *MuMIn*: version 1.43.15 [36], *arm*: version 1.10–1 [37], *lme4*: version 1.1–21 [38], and afex: version 0.28–0 [39] in R (version 3.5.1) were used for mixed effect modeling.

Further, the linear mixed model regression approach was extended to explore the relationships between fungicide use and yield losses due to seedling diseases at state and year (= temporal) scales. Models were constructed to simultaneously estimate state-specific (or year-specific, depending on the model) intercepts as well as state-specific (or year-specific, depending on the model) slopes for each individual state. As state scale yield losses and fungicide use data were classified by year, year was included as a random effect in the state-model. Similarly, as year scale yield losses and fungicide use data were classified by state, state was included as a random effect in the year-model. The regression model for state was:

$$L_{jk}=\beta_{0j}+\beta_{1j}F_{jk}+Y_{k}+e_{jk}$$

where, *L*_*jk*_ = soybean yield loss due to seedling diseases from j^th^ state in k^th^ year; *F*_*jk*_ = seed-applied fungicide use from j^th^ state in k^th^ year; *Y*_*k*_ = random effect of the k^th^ year; *β*_*0j*_ = state-specific intercept related to the j^th^ state; *β*_*1j*_ = state-specific slope related to the j^th^ state; e_jk_ = error.

Similarly, the regression model for year was:

$$L_{jk}=\beta_{0k}+\beta_{1k}F_{jk}+S_{j}+e_{jk}$$

where, *L*_*jk*_ = soybean yield loss due to seedling diseases from j^th^ state in k^th^ year; *F*_*jk*_ = seed-applied fungicide use from j^th^ state in k^th^ year; *S*_*j*_ = random effect of the j^th^ state; *β*_*0k*_ = year-specific intercept related to k^th^ year; *β*_*1k*_ = year-specific slope related to the k^th^ year; e_jk_ = error.

Analyses were performed to assess total fungicide use per unit harvest area (g/ha) and total yield loss per unit area (kg/ha), as well as total fungicide use (MT) and total yield loss (1,000 MT). In addition, the relationship between soybean production/yield and fungicide use was also explored. In all cases, same model structures (state and year, see above) were employed.

### Classification of soybean harvest, production, and yield zones

One of our goals in the present study was to investigate if the mean per hectare seed-applied fungicide use differ between the levels of yield/production/harvest zones. Moreover, we needed to conduct an exploratory multivariate analysis (see below) by incorporating yield/ production/harvest zones and per hectare seed-applied fungicide use. Therefore, using the approach outlined below, we first derived the said zone types: (i) Yield zone (1 to 4), based on USDA-NASS estimates at the state level comparing yield (MT/HA) with all state by year combinations, (ii) Production zone (1 to 4), based upon USDA-NASS estimates at the state level comparing total production (MT) with all state by year combinations, and (iii) Harvest zone (1 to 4), based upon USDA-NASS estimates at the state level comparing harvested area (HA) with all state by year combinations. We then classified 252 yield/production/harvest data points (28 states × 9 years) into zone levels where data points within the minimum to first quartile were classified as Zone 1. Similarly, data points from the first quartile to median, median to third quartile, and > third quartile were classified as zones 2, 3, and 4, respectively. Note that the zones were not solely defined based on geography, in this case state, and are a function of year (temporal scale). As such, the zone of a given data point was relative to the other data points within the database.

### Analysis of variance (ANOVA) and Factor Analysis of Mixed Data (FAMD)

Using the same specifications outlined in our previous paper [33], the main effects of harvest, production, and yield zone on the total seed-applied fungicide use (g/ha) were investigated. The full linear model that was fitted with yield zone was:

$$F_{ijk}=\mu +Z_{i}+S_{j}+Y_{k}+{\left(ZS\right)}_{ij}+{\left(ZY\right)}_{ik}+{\left(SY\right)}_{jk}+e_{ijk}$$

where, *F*_*ijk*_ is the observed total seed-applied fungicide use (in grams per hectare) for the *i*^th^ yield zone (*i* = 1–4), *j*^*t*h^ state (*j* = 1–28), and *k*^th^ year (*k* = 1–9); μ is the overall mean seed-applied fungicide use common to all yield zones; *Z*_*i*_ is the fixed effect of *i*^th^ yield zone; *S*_*j*_ is the random effect of the *j*^th^ state; *Y*_*k*_ is the random effect of the *k*^*t*h^ year; (*ZS*)_*ij*_ is the random two-way interaction effect between *i*^th^ yield zone and *j*^th^ state; (*ZY*)_*ik*_ is the random two-way interaction effect between *i*^th^ yield zone and *k*^th^ year; (*SY*)_*jk*_ is the random two-way interaction effect between *j*^th^ state and *k*^*t*h^ year; *e*_*ijk*_ is the residual. Note that the residual term e_ijk_ comprises both error and the random three-way interaction Z × S × Y. The same model structure was used for harvest and production zones.

Factor Analysis of mixed data (FAMD) was conducted using total seed-applied fungicide use (g/ha) as a quantitative variable and the year, state, region, soybean harvest zone, production zones, and yield zone as qualitative variables. FAMD is a principal component method to analyze a data set containing both qualitative and quantitative variables. As such, FAMD allows analyzing the similarity between individuals (individual data points) by taking into account mixed-variable types. With this analysis, quantitative and qualitative variables are normalized in order to balance the impact of each set of variables. Additional details on this analysis are available in our companion paper [33].

## Results

### Spatiotemporal variation of soybean seed-applied fungicide use in the United States

**Fig 1:**
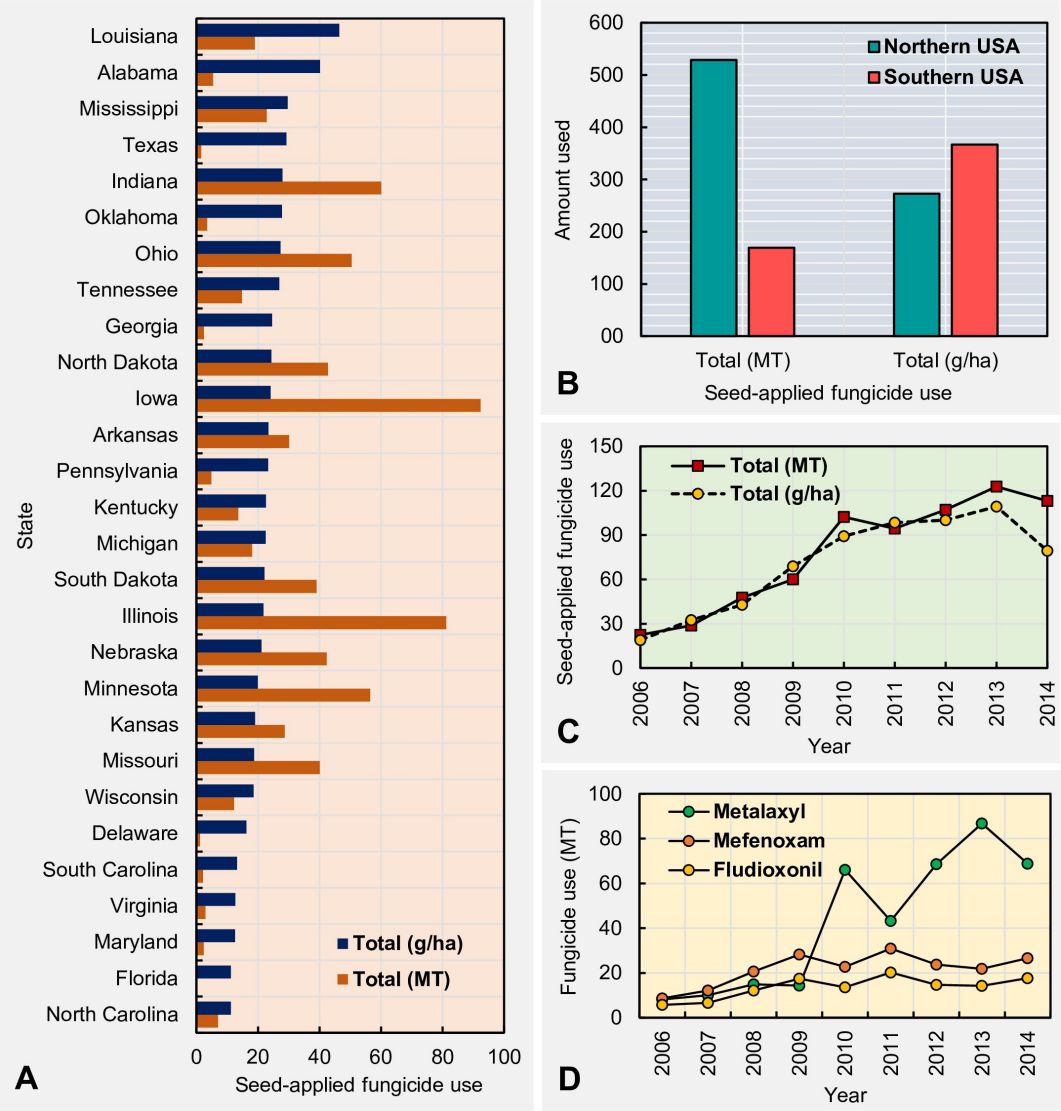
Spatiotemporal use patterns of the soybean seed-applied fungicides in the United States. (A) state-wide use of total seed-applied fungicides across nine years (2006 to 2014); (B) total seed-applied fungicide use between 2006 and 2014 by region (Northern states = Illinois, Indiana, Iowa, Kansas, Michigan, Minnesota, Nebraska, North Dakota, Ohio, Pennsylvania, South Dakota, and Wisconsin; Southern states = Alabama, Arkansas, Delaware, Florida, Georgia, Kentucky, Louisiana, Maryland, Missouri, Mississippi, North Carolina, Oklahoma, South Carolina, Tennessee, Texas, and Virgina. Fungicides included: fludioxonil, mefenoxam, and metalaxyl). Temporal fluctuation of the (C) the total seed-applied fungicide use, (D) fludioxonil, mefenoxam, and metalaxyl use across 28 states between 2006 and 2014.

Across the nine-year period from 2006 to 2014, Florida reported the lowest per hectare (11.2 g) as well as total (0.13 MT) seed-applied fungicide use. Conversely, Louisiana reported the greatest per hectare use (69.1 g) and Iowa (92.3 MT) reported the greatest total seed-applied fungicide use in the U.S. (Fig 1A). When considered regionally, the total use (MT) of seed-applied fungicides was approximately 3.1 times greater in the northern compared to southern states (Fig 1B). However, per hectare total use (g/ha) of seed-applied fungicides was approximately 1.3 times greater in the southern states compared to the northern states (Fig 1B).

The lowest and greatest seed-applied fungicide uses were recorded in 2006 and 2013, respectively (in both MT and g/ha). Despite the year-to-year variation, an increasing trend towards increasing seed-applied fungicide use was observed over time (Fig 1C). The percentage use increment from 2006 to 2013 was 447% (total fungicide use in MT) and 477% (total fungicide use in g/ha), respectively. The total uses (in MT) of fludioxonil and mefenoxam across 28 states did not dramatically fluctuate over the period between 2006 and 2014, even though metalaxyl usage fluctuated sharply within the same time period (Fig 1D). During the time period between 2006 and 2013, metalaxyl use increased approximately ten-fold.

### Mixed effect modeling of the relationship between seedling diseases associated soybean production/yield losses and seed-applied fungicide use at national and regional scales

At the national scale, where annual total soybean production loss and annual total fungicide use were considered in 1,000 MT and MT, respectively, the conditional model had a rather large R^2^ (= ***R***^***2***^_**GLMM(c)**_) value compared to that of the marginal model (= ***R***^***2***^_**GLMM(m**_) (Table 1). Fitting the full model with an added square term for fungicide use (= full quadratic model) did not improve ***R***^***2***^_**GLMM(m)**_. State had a greater variance component compared to year. Similar results were observed when annual yield loss and total fungicide use were considered in kg/ha and g/ha, respectively (Table 1).

**Table 1:** National scale mixed-effects regression modelling of the effect of seed-applied fungicide use on soybean production/yield losses due to seedling diseases from 28 soybean growing states in the United States during the time period between 2006 and 2014.

Model name	A			B		
	Null model	Full model (L)	Full model (Q)	Null model	Full model (L)	Full model (Q)
**Fixed effect**	*a* ± SE	*a* ± SE	*a* ± SE	*a* ± SE	*a* ± SE	*a* ± SE
Intercept	68.6 ± 19.2	68.6 ± 18.8	68.6 ± 18.2	72.9 ± 14.5	72.9 ± 14.8	72.9 ± 14.8
Fungicide use	-	96.7 ± 111.3	169.1 ± 119.6	-	131.4 ± 94.3	123.8 ± 97.9
Fungicide use^2^	-	-	-153.1 ± 103.1	-	-	25.5 ± 87.2
**Random effects**	VC	VC	VC	VC	VC	VC
State	7,370	7,104	6,509	4,492	4,711	4,706
Year	938	868	836	344	350	363
Residuals	6,575	6,619	6,651	5,389	5,365	5,379
***R***^***2***^_**GLMM(m)**_	-	0.002	0.012	-	0.005	0.005
***R***^***2***^_**GLMM(c)**_	-	0.547	0.530	-	0.488	0.488
**AIC**^j^	3,678	3,679	3,679	3,604	3,604	3,606
**BIC**^k^	3,693	3,697	3,701	3,619	3,623	3,628

A = relationship between annual total fungicide use (MT) and annual total production loss (1,000 MT); B = relationship between annual total fungicide use (g/ha) and annual yield loss (kg/ha); L = linear; Q = quadratic; SE = standard error; VC = variance components; States included Illinois, Indiana, Iowa, Kansas, Michigan, Minnesota, Nebraska, North Dakota, Ohio, Pennsylvania, South Dakota, Wisconsin, Alabama, Arkansas, Delaware, Florida, Georgia, Kentucky, Louisiana, Maryland, Mississippi, Missouri, North Carolina, Oklahoma, South Carolina, Tennessee, Texas, and Virginia. The national scale is a composite of all 28 states; Fungicide use^2^ = quadratic term of the fungicide use; *R*^*2*^_GLMM(*m*)_ = generalized R^2^ for marginal model; *R*^*2*^_GLMM(*c*)_ = generalized R^2^ for conditional model

^j^AIC = Akaike Information Criterion

^k^BIC = Bayesian information criterion.

Regional scale analyses conducted targeting the states defined within each region as either Northern or Southern within the U.S. also revealed significantly smaller ***R***^***2***^_**GLMM(m)**_ compared to ***R***^***2***^_**GLMM(c)**_ (S1 and S2 Tables). Inclusion of the square term for fungicide use in the full model (= full quadratic model) did not result in an improved ***R***^***2***^_**GLMM(m)**_ compared to the full linear model.

### Relationship between seed-applied fungicide use and soybean yield losses due to seedling diseases at state and year scales

Per the state-specific regression slopes, the relationship between soybean production loss due to seedling diseases (1,000 MT) and total fungicide use (MT) was significant (α = 0.1) for Illinois (*P* = 0.055), Indiana (*P* = 0.008), Michigan (*P* = 0.039), and North Carolina (*P* = 0.002) (S3 Table). Except for Michigan, slope (= regression coefficient) related to fungicide use for other states were negative. When fungicide use (g) and yield losses (kg) were considered on a per hectare basis, a significant (α = 0.1) relationship was observed for Indiana (*P* = 0.022), Michigan (*P* < 0.001), and Ohio (*P* = 0.085) (S3 Table). The slope associated with fungicide use (g) for Indiana and Ohio was negative (S3 Table).

The relationship between soybean production loss due to diseases (1,000 MT) and total fungicide use (MT) was significant (α = 0.1) for 2008 (*P* < 0.001), 2009 (*P* = 0.069), and 2014 (*P* = 0.064) (S3 Table). Nevertheless, the slope associated with fungicide use (g) for each of these years were positive (S3 Table). When losses (kg) and fungicide use (g) were considered on a per hectare basis, a significant relationship was not evident for any of the year (S3 Table).

### Mixed effect modeling of seed-applied fungicide use and soybean production/yield at national and regional scales

At the national scale, when annual total production and annual total fungicide use were considered in 1,000 MT and MT, respectively, the R^2^ for the conditional model (= ***R***^***2***^_**GLMM(c)**_) was significantly larger than that of the marginal model (= ***R***^***2***^_**GLMM(m)**_) (Table 2). The conditional model explained almost the entire (98%) observed variation for soybean production at the national scale. Inclusion of the squared term for fungicide use in the full model (= full quadratic model) did not improve the ***R***^***2***^_**GLMM(m)**_ compared to the full linear model. The variance component for state was larger than that for year. Similar results were observed when soybean yield and total annual fungicide use were considered in kg/ha and g/ha, respectively (Table 2).

**Table 2:** National scale mixed-effects modelling of the effect of seed-applied fungicide use on soybean production/yield from 28 soybean growing states in the United States during the time period between 2006 and 2014.

Model name	A			B		
	Null model	Full model (L^c^)	Full model (Q^d^)	Null model	Full model (L)	Full model (Q)
**Fixed effect**	*a* ± SE^e^	*a* ± SE	*a* ± SE	*a* ± SE	*a* ± SE	*a* ± SE
Intercept	3,101 ± 670	3,101 ± 662	3,101 ± 638	2,543 ± 113	2,542 ± 116	2,543 ± 113
Fungicide use	-	920 ± 993	2,907 ± 1114	-	-458 ± 421	-323 ± 464
Fungicide use^2^	-	-	-2,624 ± 735	-	-	-281 ± 426
**Random effects**	VC^f^	VC	VC	VC	VC	VC
State^g^	122,232,88	11,905,823	11,096,306	176,415	178,096	175,403
Year	111,065	104,048	90,748	54,664	59,348	55,605
Residuals	235,466	236,951	227,765	101,380	100,909	101,591
***R***^***2***^_**GLMM(m)**_^h^	-	0.000	0.005	-	0.002	0.002
***R***^***2***^_**GLMM(c)**_^i^	-	0.980	0.980	-	0.703	0.695
**AIC**^j^	4,033	4,034	4,023	3,729	3,729	3,731
**BIC**^k^	4,047	4,051	4,044	3,743	3,747	3,752

A = relationship between annual total fungicide use (MT) and annual total soybean production (1,000 MT); B = relationship between annual total fungicide use (g/ha) and annual yield (kg/ha)

^c^L = linear

^d^Q = quadratic

^e^SE = standard error

^f^VC = variance components

^g^States included Illinois, Indiana, Iowa, Kansas, Michigan, Minnesota, Nebraska, North Dakota, Ohio, Pennsylvania, South Dakota, Wisconsin, Alabama, Arkansas, Delaware, Florida, Georgia, Kentucky, Louisiana, Maryland, Mississippi, Missouri, North Carolina, Oklahoma, South Carolina, Tennessee, Texas, and Virginia. The national scale is a composite of all 28 states; Fungicide use^2^ = quadratic term of the fungicide use

^h^*R*^*2*^_GLMM(*m*)_ = generalized R^2^ for marginal model

^i^*R*^*2*^_GLMM(*c*)_ = generalized R^2^ for conditional model

^j^AIC = Akaike Information Criterion

^k^BIC = Bayesian information criterion.

Regional scale analyses conducted targeting the states within the Northern and Southern U.S. also revealed significantly smaller ***R***^***2***^_**GLMM(m)**_ compared to ***R***^***2***^_**GLMM(c)**_ (S4 and S5 Tables). Inclusion of the square term for fungicide use in the full model (= full quadratic model) did not improve the ***R***^***2***^_**GLMM(m)**_ compared to the full linear model.

### Relationship between seed-applied fungicide use and soybean production/yield at state and year scales

The relationship between total seed-applied fungicide use (MT) and soybean production (1,000 MT) was significant (α = 0.1) for Iowa (*P* = 0.006), Illinois (*P* = 0.098), and South Dakota (*P* = 0.045) (S6 Table). However, the slope associated with fungicide use was positive for only Illinois and South Dakota (S6 Table). When fungicide use (g) and soybean yield (kg) were considered on a per hectare basis, a significant (α = 0.1) relationship was observed for Iowa (*P* = 0.014), Kansas (*P* = 0.003), and Texas (*P* = 0.065) (S6 Table). However, for each of these states, the slope associated with fungicide use (g) was negative (S6 Table).

The relationship between total seed-applied fungicide use (MT) and soybean production (1,000 MT) was significant (α = 0.05) for all years except for 2009, 2010, and 2011 (S6 Table). However, except for years 2006 and 2014, the slopes associated with fungicide use (g) for other years were negative (S6 Table). When yield (kg) and fungicide use (g) were considered on a per hectare basis, a significant (α = 0.05) relationship was only observed for 2012, yet the slope related to fungicide use was negative (S6 Table).

### Analysis of variance (ANOVA)

**Fig 2:**
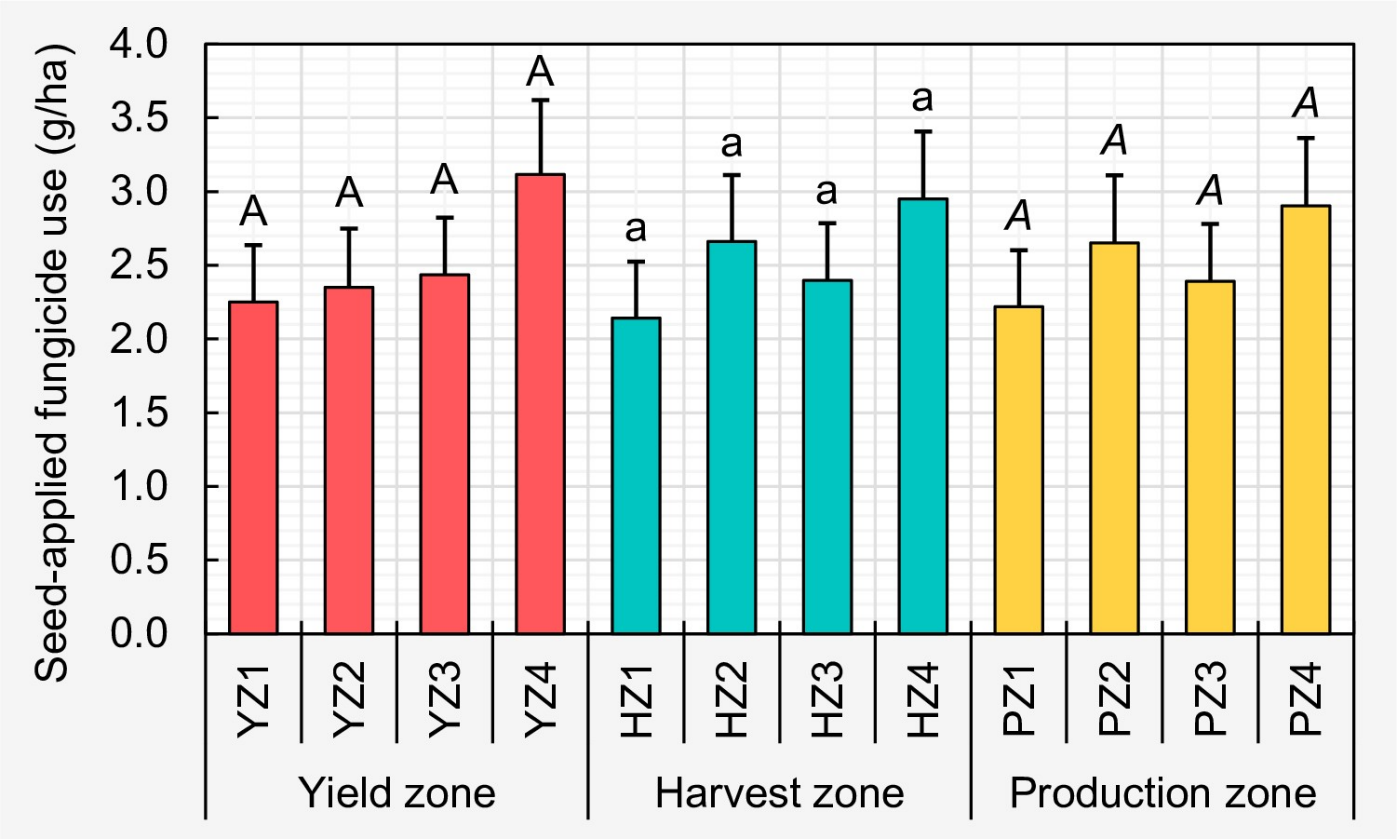
Comparison of the mean per hectare seed-applied fungicide use (in g) among yield/harvest/production zones. Within each zone type, means followed by a common letter are not significantly different after adjustment for multiple comparisons using Tukey-Kramer test at the 5% level of significance. Error bars represent standard errors. The fungicides included: fludioxonil, mefenoxam, and metalaxyl. The selected fungicides are effective against Fusarium wilt, Phytophthora root and stem rot, and seedling diseases caused by *Fusarium*, *Phomopsis* spp., *Pythium*, and *Rhizoctonia solani*.

ANOVA showed a non-significant main effect of yield zone, harvest zone, and production zone (*α* = 0.05) on seed-applied fungicide use. Consequently, mean per hectare seed-applied fungicide use (in g) was not significantly different among each of the previously defined levels of yield/harvest/production zones (Fig 2).

### Factor analysis of mixed data (FAMD)

**Fig 3:**
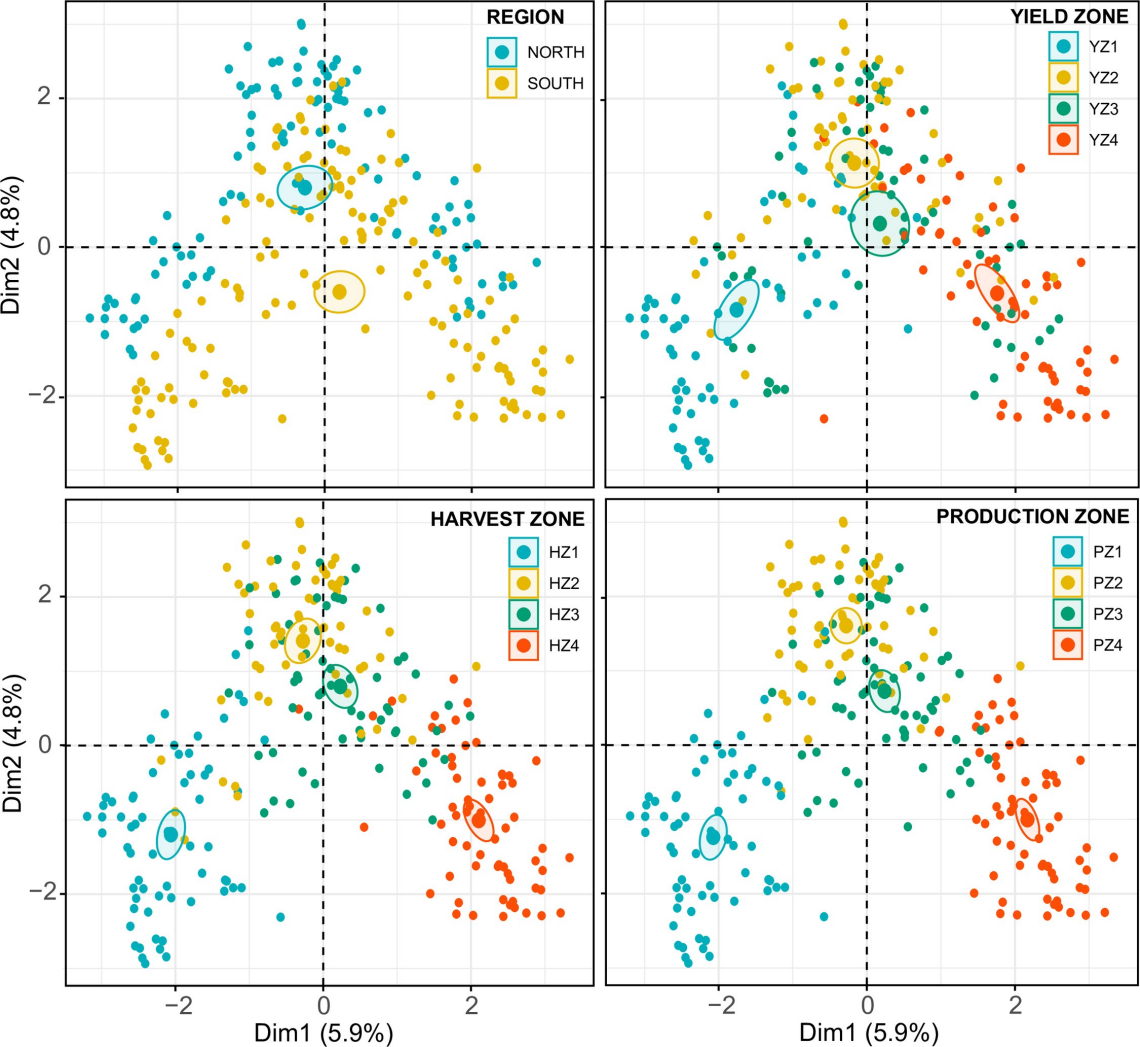
Factor maps generated through factor analysis of mixed data (FAMD) approach showing the variance maximizing distribution pattern of data points (n = 252) in the map space with their clustering patterns based upon region (n = 2), and yield/harvest/production zones (n = 4 in each case). Yield/Harvest/Production zones = represent four levels (zone 1 to 4) based on the quartiles within a database containing 252 yield (kg/ha)/harvest area (ha)/production (MT) data points (252 = 9 years × 28 states). Within this database, data points from the minimum to the first quartile were classified as zone 1. Similarly, data points from the first quartile to median, median to the third quartile, and > third quartile were respectively classified as zones 2, 3, and 4. Fungicides included: fludioxonil, mefenoxam, and metalaxyl which are effective against Fusarium wilt, Phytophthora root and stem rot, and seedling diseases caused by, *Fusarium*, *Phomopsis* spp., *Pythium*, and *Rhizoctonia solani*.

The variance maximizing data point distribution in the factor map did not reveal a clear clustering pattern based upon state, year, or yield zone. However, a clear clustering was observed based upon region, harvest zone, and production zone (Fig 3). Factor maps showed that yield/harvest/production zones 2 and 3 clustered closely while zones 1 and 4 were distantly clustered.

## Discussion

An increasing number of inputs including seed-applied fungicides have become an integral component of farmer’s soybean management strategies to achieve maximum yield [40, 41]. Over the past two decades the cost of soybean seed has increased as a result of the increasing costs associated with herbicide trait technologies and the technology fees associated with those herbicide traits. More specifically, since 1975, the cost of soybean seed has increased $3.37 to $23.79 per hectare in 2016, a 7-fold increase [42]. To offset those costs, farmers have attempted to decrease seeding rates and therefore have increased the use of seed-applied fungicides as a method to protect the seed planted; a type of “insurance” has been a common term applied to the use of seed-applied fungicides [20, 40, 43]. Seed-applied fungicides therefore serve as a method to reduce overall production costs by reducing the likelihood of a replant situation which increases overall soybean production costs if and when stand failures result in the need for purchasing additional seed and then the subsequent costs associated with planting (e.g., fuel, labor, equipment depreciation) [32, 42, 44]. As a result of increasing soybean production costs and decreasing seeding rates to offset increased seed costs, seed-applied fungicide use has significantly increased over the past two decades. More specifically, Munkvold [45] reported that some seed companies estimated that in 1996, nearly 8% of soybean seed was treated with a seed-applied fungicide while in 2008 estimates of total seed receiving a seed-applied fungicide had reached 30%. In addition, the most recent estimates of soybean seed having received at least one fungicide prior to planting suggest that 60–75% are treated [44, 46].

In the current study, the regional scale data revealed that the total seed-applied fungicide use (in MT) was greater in the northern states compared to the southern states. The associated differences between the northern and southern U.S. may be predominantly attributable to the greater land use for soybean production in the north compared to the south. In general, and over the period of time in question, approximately three times more land area was devoted to soybean production in the northern states (12 states) compared to the southern states (16 states). However, the per hectare use (g) of seed-applied fungicides was greater in the southern compared to the northern region. Greater per hectare use of seed-applied fungicides in the southern U.S. may be associated with prolonged periods of conducive environment for the development of seedling-associated diseases. However, one additional factor likely contributes to greater seedling disease occurrence probabilities due to factors such as an extended period of soybean planting (March to July) and seedling disease conducive soil conditions. In the southern U.S., the soil environment can greatly impact the incidence of seedling-associated diseases that result from extended periods of excessive soil moisture and planting into soils early in the season when marginal temperatures occur [47]. As such, farmers in this region generally opt to use seed-applied fungicides with greater frequency to reduce the probabilities of poor seed emergence or reduced stands that can in some cases necessitate a replant situation which increases production costs and can result in reduced yield potential by altering the planting date. Moreover, the different crop rotations used in the southern U.S. could also contribute to the increased adoption of seed-applied fungicides as a form of “cheap insurance”. For example, crops are planted within a narrow window, and a farmer may need to plant the soybean crop before moving onto another commodity. Therefore, the seed-applied fungicide may reduce the likelihood of having to go back to planting a previous crop which can not only delay the soybean crop, but a crop that is historically planted a little later than soybean, such as cotton.

One of the primary objectives of our study was to investigate the relationship between fungicide use and soybean production/yield loss due to seedling diseases using data from different soybean growing states and years. Given that soybean production/yield loss data and fungicide use data were classified by year and state, we deployed a linear mixed model approach to model the effect of seed-applied fungicide use on soybean yield losses due to seedling diseases at a national and regional scale by specifying state and year as random effects. The difference of generalized R^2^ values between conditional (fixed and random effects; fungicide use + state + year = ***R***^***2***^_**GLMM(c)**_) and marginal (only fixed effects; fungicide use = ***R***^***2***^_**GLMM(m)**_) models were large and ***R***^***2***^_**GLMM(c)**_ **>>> *R***^***2***^_**GLMM(m)**_. Therefore, national and regional scale analyses did not establish strong relationship between seed-applied fungicide use and seedling diseases associate soybean production/yield loss. However, as the addition of random effects (state and year) into the models resulted in significantly larger R^2^, we focused on individual state and year to further explore the trends.

As mentioned in the methods section, to obtain state and temporal scale insights, we used total fungicide use (i.e. summation of the three fungicide active ingredients) as the regressor variable within the mixed model regression framework. The use of the mixed model version of the of multiple linear regression to make inferences on the utility of individual active ingredients in reducing yield losses due to seedling diseases was not appeared to be appropriate due to three reasons. (i) it is not legitimate to regress the total yield lose due to seedling diseases (the regressand) against individual active ingredients as each individual active ingredient does not necessarily effective in controlling the yield loss due to all seedling diseases considered in the study, (ii) some active ingredients are effective against more than one seedling diseases. Therefore, yield loss reduction due to a given disease (the regressand) cannot be attributable to a single active ingredient. As such, yield loss due to a single disease cannot be regressed against individual active ingredients, (iii) although the pesticide database contains information on fungicide use in terms of active ingredients, that does not necessarily mean that active ingredients were used by farmers as stand-alone products. For instance, while commercial products such as Allegiance LS^®^ (Metalaxyl), Apron XL LS^®^ (Mefenoxam), and Maxim 4FS^®^ (Fludioxonil) are stand-alone fungicides, many trademarks/products contain a pre-mix of active ingredients. For example, Apron Maxx^®^, Cruiser Maxx^®^, and Warden RTA^®^ are some of the heavily used pre-mixed products, consist of a combination of Fludioxonil and Mefenoxam. Importantly, it is possible that the interaction effect between active ingredients of pre-mixed products play a role in disease suppression (thus the magnitude of the yield loss). Although the representative interaction terms for different active ingredients can be included in the multiple regression, with the data available in the pesticide data base, it is impossible to know what proportions of each active ingredient was used as stand-alone and pre-mixed products. Therefore, fitting multiple regression models using individual active ingredients and their combinations as regressor variables is not realistic with the type of data that we deal with. As such, the only sensible resort is to regress the total yield loss due to all diseases against the summation of individual active ingredients using the mixed effect version of simple linear regression.

Analyses conducted at the state level showed significant and negative relationship between soybean production loss (1,000 MT) and seed-applied fungicide use (MT) for Illinois, Indiana, and North Carolina, which are some of the major soybean growing states in the U.S. When the relationship was considered with per hectare basis data, yield loss (kg) was significantly and negatively related with seed-applied fungicide use (kg) for Indiana and Ohio. As such, seed-applied fungicide use appeared to be a useful tool to mitigate seedling diseases associated soybean yield loses in said states. The absence of significant and negative relationships between fungicide use and soybean production/yield loss for vast majority of the states can be partly due to the nature of data that was used for this study. For example, the yield loss data used in this study were all estimates, made by soybean disease experts including Extension pathologists based on their field observations. Although expert’s estimations were strengthened by using Padwick’s calculation in computing the overall loss with a standardized method enabling estimates of impact due to seedling diseases and among types of soybean diseases, the possibility that computed yield losses are different from actual yield losses cannot be understated. Furthermore, although the fungicide use data available in the Pesticide National Synthesis Project webpage is not based on sales data but actual use data, they are still estimated values. The methods used in estimating the fungicide use are robust but still may differ from the actual use values. Although the majority of soybean hectares in the U.S. are planted with seed having received a pesticide/fungicide, reliable data on the actual use of seed-applied pesticides/fungicides are generally sparse [48].

The total soybean production/yield (1,000 MT, kg/ha) did not appear to increase with the total seed-applied fungicide use (MT, g/ha) for the majority of the soybean producing states except Illinois and South Dakota. Our findings are in agreement with previous reports; however, such reports only address the effectiveness of seed-applied fungicides on a much smaller geographic scale, generally at a specific location. For example, the research from across the U.S. has generally failed to show consistent benefits from seed-applied fungicides. Bradley et al. [31] reported that metalaxyl-applied seed treatment resulted in increased soybean stands in one year of a two-year study, but not yield while Cox et al. [32] reported no differences in both stand establishment and yield between non-treated soybean seed and seed-applied fungicide treatments. Schulz and Thelen [28] reported that seed treated with metalaxyl and fludioxonil increased soybean yield in only three of 16 site-years yet decreased yield in two of 16 site-years. In the specific situations where yield was observed to provide a positive return, the soil conditions were wet and cool early-season, which coincides with the expectations for seed-applied fungicides. With multi-environmental investigations, Gasper et al. [44, 49] reported that the effect of seed-applied fungicides on plant stand and yield were environment specific.

Results of the ANOVA revealed that mean seed-applied fungicide usage (g/ha) was not significantly different among the four distinct zones within each yield/harvest/production zone category. The current results as they relate to zone indicated that despite the apparent soybean yield/production differences between different zones (i.e., zone 4 = greatest yield/production, zone 1 = lowest yield/production), similar amounts of seed-applied fungicides are being used across all zones. Therefore, factors other than seed-applied fungicides likely were contributing to the greater yield/production associated with zone 4. On the other hand, it is possible that a greater number of farmers in low yield/harvest/production zones use soybean seeds treated with fungicides compared to non-fungicide-treated seeds with the expectation of a yield increase or reducing the potential costs associated with a replant situation.

The aim of the current investigation was to examine the patterns of seed-applied fungicide use estimates and its relationship with estimated soybean yield losses due to seedling diseases at broader spatial (national/regional/state) scales within the U.S. It is important to note that the trends that we observed at broader geographic scales may or may not essentially correlate with farm-scale trends, especially since the chemical concentrations assessed were based on estimates of use of each active ingredient within a given geographic boundary (i.e., state). For example, the absence of a significant and negative relationship between fungicide use and yield losses at the state level does not necessarily mean that seed-applied fungicides failed to reduce yield losses as a result of seedling diseases across the planted area within that state. Therefore, the current paper does not intend to guide fungicide use decision making on a farm-scale. An individual farmer may decide on seed treatment use based on several field-level factors such as planting date, which greatly determines the vulnerability of seed/seedlings to certain soilborne pathogens, cultivar susceptibility, and long-term disease history in a specific field location. Farmers should also pay enough attention on manipulating plant populations and row spacing, appropriate tillage and crop rotation practices, suitable abiotic stress management measures such as proper fertility and irrigation regimes to reduce the risk of seedling disease incidence and severity as well as maximize the attainable yield.

While in our previous article [33], we elucidated the relationship between foliar fungicide use and soybean yield losses due to foliar diseases, the current paper examined the relationship between seed-applied fungicide use and soybean yield losses due to seedling diseases. In both papers, we applied a similar analytical/methodological approach given the similarity in the type of questions that were under study, with the recognition that the primary topics of interest are contrastingly different between two parts (foliar vs seed-applies fungicides). The use of mixed model approach in both articles allowed incorporation random effects into the national/regional/state/temporal scale models. This in turn helped determining the statistically prudent relationships between foliar/seed-applied fungicide use and soybean yield losses due to foliar/seedling diseases, otherwise would have been modified by the random factors. Results from both articles revealed general lack of fit between fungicide use and yield loss/production at national and regional scales as well as at state scale for vast majority of the soybean growing states. Furthermore, the variation of yield loss as well as production were predominantly explained by the state and year rather than the fungicide use.

Previously published landscape level fungicide studies do not always emphasize the usefulness of foliar/seed-applied fungicide use to mitigate disease-associate yield losses. In that sense, the general lack of strong negative relationships that were evident through our work between fungicide use and soybean yield losses was not that surprising. However, we do not undermine the possible contribution of the nature of the data towards our observations. We should note that, although scientifically robust approaches were deployed, both fungicide use and yield loss data were estimated values and can therefore be different from actual data, which does not currently exist at the scale of our current work. This article, along with our previously published companion article, are the first to investigate relationships between estimated foliar and seed-applied fungicide use estimated soybean yield losses due to foliar and seedling diseases in the U.S. at national, regional, and state levels and provide useful insights to policy makers and researchers conducting field-scale fungicide efficacy trials in soybean and other agriculturally important crops.

## Supporting information

S1 Table Mixed-effects modelling of the effect of seed-applied fungicide use on soybean yield losses due to foliar diseases from soybean growing states in the northern region of the United States during 2006–2014 period. (DOCX)

S2 Table Mixed-effects modelling of the effect of seed-applied fungicide use on soybean yield losses due to foliar diseases from soybean growing states in the southern region of the United States during 2006–2014 period. (DOCX)

S3 Table Mixed-effects modelling of the effect of seed-applied fungicide use on soybean production/yield loss for 28 states in the United States and at temporal scale during 2006–2014 period. (XLSX)

S4 Table Mixed-effects modelling of the effect of seed-applied fungicide use on soybean production/yield from soybean growing states in the northern region of the United States during 2006–2014 period. (DOCX)

S5 Table Mixed-effects modelling of the effect of seed-applied fungicide use on soybean production/yield from soybean growing states in the southern region of the United States during 2006–2014 period. (DOCX)

S6 Table Mixed-effects modelling of the effect of seed-applied fungicide use on soybean production/yield for 28 states in the United States and at temporal scale during 2006–2014 period. (XLSX)
