# Supplementary material for: Modeling the relationship between estimated fungicide use and disease-associated yield losses of soybean in the United States II: Seed-applied fungicides vs seedling diseases
Source: PLoS One. 2020 Dec 28;15(12):e0244424. doi: 10.1371/journal.pone.0244424 (PMC7769478; doi:10.1371/journal.pone.0244424)
Supplement: S1 Table — (DOCX) [file pone.0244424.s001.docx]

**Supplementary table 1.** Regional scale mixed-eﬀects modeling of the eﬀect of seed-applied fungicide use on soybean production/yield losses due to seedling diseases from soybean growing states in the northern United States during the time period between 2006 and 2014.

|  | A^a^ | | |  | B^b^ | | |
| --- | --- | --- | --- | --- | --- | --- | --- |
| Model name | Null model | Full model (L^c^) | Full model (Q^d^) |  | Null model | Full model (L) | Full model (Q) |
| **Fixed effect** | *a* ± SE^e^ | *a* ± SE | *a* ± SE |  | *a* ± SE | *a* ± SE | *a* ± SE |
| Intercept | 192.1 ± 32.6 | 192.1 ± 33.9 | 192.1 ± 30.2 |  | 122.4 ± 21.4 | 122.4 ± 21.4 | 122.4 ± 21.0 |
| Fungicide use | - | -120.1 ± 182.4 | -54.3 ± 172.6 |  | - | -1.9 ± 100.1 | -3.0 ± 99.8 |
| Fungicide use^2^ | - | - | -303.1 ± 156.2 |  | - | - | -130.2 ± 98.0 |
|  |  |  |  |  |  |  |  |
| **Random effects** | VC^f^ | VC | VC |  | VC | VC | VC |
| State^g^ | 10,432 | 11,271 | 8,840 |  | 4,485 | 4,484 | 4,306 |
| Year | 244 | 408 | 67 |  | - | - | - |
| Residuals | 17,703 | 17,551 | 17,789 |  | 8,930 | 9,022 | 8,986 |
|  |  |  |  |  |  |  |  |
| ***R^2^*_GLMM(_*_m_*_)_**^h^ | - | 0.005 | 0.032 |  | - | 0.000 | 0.012 |
| ***R^2^*_GLMM(_*_c_*_)_**^i^ | - | 0.402 | 0.355 |  | - | 0.332 | 0.332 |
| **AIC**^j^ | 1,393 | 1,395 | 1,393 |  | 1,314 | 1,316 | 1,317 |
| **BIC**^k^ | 1,404 | 1,408 | 1,409 |  | 1,323 | 1,327 | 1,330 |

^a^ A = relationship between annual total fungicide use (MT) and annual total production loss (1,000 MT).

^b^ B = relationship between annual total fungicide use (g/ha) and annual yield loss (kg/ha).

^c^ L = linear.

^d^ Q = quadratic.

^e^ SE = standard error.

^f^ VC = variance components.

^g^ States in the northern region included Illinois, Indiana, Iowa, Kansas, Michigan, Minnesota, Nebraska, North Dakota, Ohio, Pennsylvania, South Dakota, and Wisconsin. The northern regional scale is a composite of all 12 states.

^h^ *R^2^*_GLMM(_*_m_*_)_ = generalized R^2^ for marginal model.

^i^ *R^2^*_GLMM(_*_c_*_)_ = generalized R^2^ for conditional model.

^j^AIC = Akaike Information Criterion.

^k^ BIC = Bayesian Information Criterion.

Note that due to model overfitting (singularity), “Year” was excluded when analysis was conducted to explore the relationship between annual total fungicide use in g/ha and annual yield loss in kg/ha.
