# Supplementary material for: Modeling the relationship between estimated fungicide use and disease-associated yield losses of soybean in the United States II: Seed-applied fungicides vs seedling diseases
Source: PLoS One. 2020 Dec 28;15(12):e0244424. doi: 10.1371/journal.pone.0244424 (PMC7769478; doi:10.1371/journal.pone.0244424)
Supplement: S2 Table — (DOCX) [file pone.0244424.s002.docx]

**Supplementary table 2.** Regional scale mixed-eﬀects modeling of the eﬀect of seed-applied fungicide use on soybean production/yield losses due to seedling diseases from soybean growing states in the southern United States during the time period between 2006 and 2014.

|  | A^a^ | | |  | B^b^ | | |
| --- | --- | --- | --- | --- | --- | --- | --- |
| Model name | Null model | Full model (L^c^) | Full model (Q^d^) |  | Null model | Full model (L) | Full model (Q) |
| **Fixed effect** | *a* ± SE^e^ | *a* ± SE | *a* ± SE |  | *a* ± SE | *a* ± SE | *a* ± SE |
| Intercept | 14.6 ± 7.0 | 14.6 ± 6.4 | 14.6 ± 6.5 |  | 13.0 ± 3.3 | 13.0 ± 3.3 | 13.0 ± 3.3 |
| Fungicide use | - | 83.7 ± 68.6 | 81.9 ± 69.5 |  | - | -5.6 ± 16.2 | -5.2 ± 16.3 |
| Fungicide use^2^ | - | - | 7.0 ± 63.9 |  | - | - | -7.1 ± 15.6 |
|  |  |  |  |  |  |  |  |
| **Random effects** | VC^f^ | VC | VC |  | VC | VC | VC |
| State^g^ | 409 | 289 | 300 |  | 148 | 149 | 147 |
| Year | - | - | - |  | - | - | - |
| Residuals | 3,352 | 3,408 | 3,426 |  | 218 | 220 | 221 |
|  |  |  |  |  |  |  |  |
| ***R^2^*_GLMM(_*_m_*_)_**^h^ | - | 0.013 | 0.012 |  | - | 0.000 | 0.001 |
| ***R^2^*_GLMM(_*_c_*_)_**^i^ | - | 0.090 | 0.092 |  | - | 0.405 | 0.399 |
| **AIC**^j^ | 1,594 | 1,595 | 1,597 |  | 1,221 | 1,222 | 1,224 |
| **BIC**^k^ | 1,603 | 1,607 | 1,612 |  | 1,229 | 1,234 | 1,239 |

^a^ A = relationship between annual total fungicide use (MT) and annual total production loss (1,000 MT).

^b^ B = relationship between annual total fungicide use (g/ha) and annual yield loss (kg/ha).

^c^ L = linear.

^d^ Q = quadratic.

^e^ SE = standard error.

^f^ VC = variance components.

^g^ States in the southern region included Alabama, Arkansas, Delaware, Florida, Georgia, Kentucky, Louisiana, Maryland, Mississippi, Missouri, North Carolina, Oklahoma, South Carolina, Tennessee, Texas, and Virginia. The southern regional scale is a composite of all 16 states.

^h^ *R^2^*_GLMM(_*_m_*_)_ = generalized R^2^ for marginal model.

^i^ *R^2^*_GLMM(_*_c_*_)_ = generalized R^2^ for conditional model.

^j^AIC = Akaike Information Criterion.

^k^ BIC = Bayesian Information Criterion.

Note that due to model overfitting (singularity), “Year” was excluded in the analyses.
