# Supplementary material for: Modeling the relationship between estimated fungicide use and disease-associated yield losses of soybean in the United States II: Seed-applied fungicides vs seedling diseases
Source: PLoS One. 2020 Dec 28;15(12):e0244424. doi: 10.1371/journal.pone.0244424 (PMC7769478; doi:10.1371/journal.pone.0244424)
Supplement: S4 Table — (DOCX) [file pone.0244424.s004.docx]

**Supplementary table 4.** Regional scale mixed-eﬀects modeling of the eﬀect of seed-applied fungicide use on soybean production/yield from soybean growing states in the northern United States during the time period between 2006 and 2014.

|  | A^a^ | | |  | B^b^ | | |
| --- | --- | --- | --- | --- | --- | --- | --- |
| Model name | Null model | Full model (L^c^) | Full model (Q^d^) |  | Null model | Full model (L) | Full model (Q) |
| **Fixed effect** | *a* ± SE^e^ | *a* ± SE | *a* ± SE |  | *a* ± SE | *a* ± SE | *a* ± SE |
| Intercept | 5,662 ± 1,118 | 5,662 ± 1,124 | 5,662 ± 1,078 |  | 2,838 ± 135 | 2,838 ± 134 | 2,838 ± 134 |
| Fungicide use | - | -240 ± 1,276 | 1,881 ± 1,451 |  | - | 314 ± 427 | 275 ± 457 |
| Fungicide use^2^ | - | - | -2,439 ± 898 |  | - | - | 67 ± 313 |
|  |  |  |  |  |  |  |  |
| **Random effects** | VC^f^ | VC | VC |  | VC | VC | VC |
| State^g^ | 14,592,088 | 14,722,106 | 13,573,217 |  | 179,908 | 178,256 | 177,964 |
| Year | 279,863 | 292,042 | 252,108 |  | 24,318 | 22,152 | 23,143 |
| Residuals | 353,811 | 356,076 | 339,354 |  | 56,940 | 57,711 | 58,170 |
|  |  |  |  |  |  |  |  |
| ***R^2^*_GLMM(_*_m_*_)_**^h^ | - | 0.000 | 0.006 |  | - | 0.004 | 0.003 |
| ***R^2^*_GLMM(_*_c_*_)_**^i^ | - | 0.977 | 0.976 |  | - | 0.777 | 0.776 |
| **AIC**^j^ | 1,783.2 | 1,785.2 | 1,779.8 |  | 1,551.3 | 1,552.7 | 1,554.7 |
| **BIC**^k^ | 1,793.9 | 1798.6 | 1,795.9 |  | 1,562.0 | 1,566.1 | 1,570.8 |

^a^ A = relationship between annual total fungicide use (MT) and annual total soybean production (1,000 MT).

^b^ B = relationship between annual total fungicide use (g/ha) and annual yield (kg/ha).

^c^ L = linear.

^d^ Q = quadratic.

^e^ SE = standard error.

^f^ VC = variance components.

^g^ States in northern region included Illinois, Indiana, Iowa, Kansas, Michigan, Minnesota, Nebraska, North Dakota, Ohio, Pennsylvania, South Dakota, and Wisconsin. The northern regional scale is a composite of all 12 states.

^h^ *R^2^*_GLMM(_*_m_*_)_ = generalized R^2^ for marginal model.

^i^ *R^2^*_GLMM(_*_c_*_)_ = generalized R^2^ for conditional model.

^j^AIC = Akaike Information Criterion.

^k^ BIC = Bayesian Information Criterion.
