# Supplementary material for: Modeling the relationship between estimated fungicide use and disease-associated yield losses of soybean in the United States II: Seed-applied fungicides vs seedling diseases
Source: PLoS One. 2020 Dec 28;15(12):e0244424. doi: 10.1371/journal.pone.0244424 (PMC7769478; doi:10.1371/journal.pone.0244424)
Supplement: S5 Table — (DOCX) [file pone.0244424.s005.docx]

**Supplementary table 5.** Regional scale mixed-eﬀects modeling of the eﬀect of seed-applied fungicide use on soybean production/yield from soybean growing states in the southern United States during the time period between 2006 and 2014.

|  | A^a^ | | |  | B^b^ | | |
| --- | --- | --- | --- | --- | --- | --- | --- |
| Model name | Null model | Full model (L^c^) | Full model (Q^d^) |  | Null model | Full model (L) | Full model (Q) |
| **Fixed effect** | *a* ± SE^e^ | *a* ± SE | *a* ± SE |  | *a* ± SE | *a* ± SE | *a* ± SE |
| Intercept | 1,180 ± 372 | 1,180 ± 349 | 1,180 ± 338 |  | 2,321 ± 129 | 2,321 ± 131 | 2,321 ± 126 |
| Fungicide use | - | 1,366 ± 539 | 1,923 ± 638 |  | - | -235 ± 410 | -41 ± 436 |
| Fungicide use^2^ | - | - | -676 ± 434 |  | - | - | -519 ± 413 |
|  |  |  |  |  |  |  |  |
| **Random effects** | VC^f^ | VC | VC |  | VC | VC | VC |
| State^g^ | 2,127,582 | 1,875,283 | 1,756,437 |  | 67,102 | 68,437 | 65,250 |
| Year | 46,408 | 38,213 | 35,717 |  | 105,789 | 108,645 | 99,721 |
| Residuals | 84,705 | 83,403 | 83,446 |  | 106,349 | 106,544 | 107,166 |
|  |  |  |  |  |  |  |  |
| ***R^2^*_GLMM(_*_m_*_)_**^h^ | - | 0.006 | 0.015 |  | - | 0.001 | 0.007 |
| ***R^2^*_GLMM(_*_c_*_)_**^i^ | - | 0.958 | 0.956 |  | - | 0.625 | 0.609 |
| **AIC**^j^ | 2,154.7 | 2,150.4 | 2,149.9 |  | 2,136.6 | 2,138.3 | 2,138.6 |
| **BIC**^k^ | 2,166.5 | 2,165.2 | 2,167.7 |  | 2,148.5 | 2,153.1 | 2,156.5 |

^a^ A = relationship between annual total fungicide use (MT) and annual total soybean production (1,000 MT).

^b^ B = relationship between annual total fungicide use (g/ha) and annual yield (kg/ha).

^c^ L = linear.

^d^ Q = quadratic.

^e^ SE = standard error.

^f^ VC = variance components.

^g^ States in southern region included Alabama, Arkansas, Delaware, Florida, Georgia, Kentucky, Louisiana, Maryland, Mississippi, Missouri, North Carolina, Oklahoma, South Carolina, Tennessee, Texas, and Virginia. The southern regional scale is a composite of all 16 states.

^h^ *R^2^*_GLMM(_*_m_*_)_ = generalized R^2^ for marginal model.

^i^ *R^2^*_GLMM(_*_c_*_)_ = generalized R^2^ for conditional model.

^j^AIC = Akaike Information Criterion.

^k^ BIC = Bayesian Information Criterion.
